# Controversies in Pediatric Perioperative Airways

**DOI:** 10.1155/2015/368761

**Published:** 2015-11-22

**Authors:** Jozef Klučka, Petr Štourač, Roman Štoudek, Michaela Ťoukálková, Hana Harazim, Martina Kosinová

**Affiliations:** ^1^Department of Pediatric Anesthesiology and Intensive Care Medicine, Medical Faculty of Masaryk University and University Hospital Brno, Cernopolni 9, 613 00 Brno, Czech Republic; ^2^Department of Anesthesiology and Intensive Care Medicine, Medical Faculty of Masaryk University and University Hospital Brno, Jihlavska 20, 625 00 Brno, Czech Republic

## Abstract

Pediatric airway management is a challenge in routine anesthesia practice. Any airway-related complication due to improper procedure can have catastrophic consequences in pediatric patients. The authors reviewed the current relevant literature using the following data bases: Google Scholar, PubMed, Medline (OVID SP), and Dynamed, and the following keywords: Airway/s, Children, Pediatric, Difficult Airways, and Controversies. From a summary of the data, we identified several controversies: difficult airway prediction, difficult airway management, cuffed versus uncuffed endotracheal tubes for securing pediatric airways, rapid sequence induction (RSI), laryngeal mask versus endotracheal tube, and extubation timing. The data show that pediatric anesthesia practice in perioperative airway management is currently lacking the strong evidence-based medicine (EBM) data that is available for adult subpopulations. A number of procedural steps in airway management are derived only from adult populations. However, the objective is the same irrespective of patient age: proper securing of the airway and oxygenation of the patient.

## 1. Introduction

Managing the airway is crucial and the cornerstone of pediatric anesthesia. Airways in children are developing and changing during growth. They differ from adult airways in several aspects: they are narrower and the risk of swelling is greater and this can lead to increased airway resistance and breathing in a spontaneously breathing child in the postoperative period. The narrowest part of the airway is located at the level of cricoid cartilage in contrast to adults where we can choose the ETT (endotracheal tube) depending on the space between the vocal cords. The results of several MRI (magnetic resonance imaging) studies however indicate that the narrowest part can be the glottis [1]. To the best of our knowledge, this is the first airway management review article which summarizes all current controversies related to pediatric airway management.

## 2. Methods

We searched https://scholar.google.com, http://www.ncbi.nlm.nih.gov/pubmed/, Medline (OVID SP), and Dynamed for keywords: Airway/s, Children, Pediatric, Difficult Airways, and Controversies. We searched for data published between 2000 and 6/2015. After data collection, we identified several controversies related to pediatric airway management: difficult airway prediction, difficult airway management, cuffed versus uncuffed endotracheal tubes for securing pediatric airway, RSI (rapid sequence induction) in pediatric anesthesia, laryngeal mask (LM) versus endotracheal tube, and extubation timing.

## 3. Results and Discussion

The review is derived from the data of review articles (*n* = 35), prospective trials (*n* = 6), guidelines (*n* = 3), retrospective trials (*n* = 1), and meta-analysis (*n* = 1). The paucity of randomized controlled trials and meta-analyses included are a limitation of the paper but this is due to the lower number of randomized controlled trials (RCTs) related to the topic.

### 3.1. Difficult Airway Prediction

The airway management should be planned and the anesthesiologist should have a back-up plan for the scenario “when things can go wrong.” The airway evaluation needs to include the patient's medical history: birth complications, history of trauma, previous surgery, and airway management during previous anesthesia. During the clinical examination, the anesthesiologist should seek for signs of stridor, dysphonia, swallowing disorders, difficulty in breathing, difficulty in speaking, and hoarseness. There are currently a number of difficult airway predictors, but their sensitivity and specificity vary in clinical practice. The predictors with good performance are mandibular protrusion, Mallampati's classification, movement of atlantooccipital joint [2], reduced mandibular space, and increased tongue thickness [3]. Other published risk factors are age less than one year, ASA (American Society of Anesthesiologists) status III and IV, obesity (BMI, body mass index, ≥35), and patients undergoing oromaxillofacial, ENT (ear, nose, and throat), and cardiac surgery [4, 5]. The thyromental distance can be used for difficult airway prediction: the normal value should be at least 3 finger breadths (patient's 3 finger breadths) [6]. The reported incidence of difficult airway in pediatric population however is lower than that for adults and predictable in the majority [7]. Unexpected difficult face mask ventilation (inadequate mask seal, excessive gas leak, or excessive resistance) in children varies from 2.8 to 6.6% [8] and the incidence of difficult endotracheal intubation (defined as Cormack and Lehane greater than grade 3) varies between 0.06% and 1.34% [4, 9, 10]. Difficult airway should be anticipated in several congenital syndromes: Pierre robin sequence, Goldenhar syndrome, Treacher Collins syndrome, Apart syndrome, Hunter and Hurlet syndrome, Backwith-Wiedermann syndrome, Freeman-Sheldon syndrome, Down syndrome, Klippel-Fail syndrome, Hallermann-Streiff syndrome, Arthrogryposis, Cri-du-chat syndrome, Edwards syndrome, and Fibrodysplasia ossificans progressiva [11–13].

Perioperative respiratory complications still remain one of the main causes of pediatric perioperative morbidity [14] and are the second most common cause of perioperative cardiac arrest in children [15]. In clinical practice, it is advisable to combine predictors with good performance and clinical examination to predict possible difficult airway. The best way to avoid airway-related complications is regular training for the cannot intubate cannot ventilate (CICV) scenario and stepwise difficult airway protocol implementation in routine clinical practice [7].

### 3.2. Expected Difficult Airway

In case of elective surgery, the pediatric patient with known or expected difficult airway should be treated in a tertiary center [7]. Currently, there are no guidelines on how to proceed in this scenario and the majority of anesthesiologists attempt to preserve the patient's spontaneous ventilation during the period of airway securing [8]. In adulthood, the recommended clinical practice is relatively clear and the fibreoptic awake intubation with spontaneous ventilation under local anesthesia or under mild sedation can be considered as a golden standard in case of expected difficult airway [9]. This is not easy to adopt in children due to lack of cooperation of pediatric patients and, in the vast majority of pediatric patients, the airways can be managed only after anesthesia induction or under deep sedation [16]. There are conflicting data on the role of muscle relaxants in the case of expected difficult airway in children. Some authors permit their use in case of possible facemask or supraglottic device ventilation with the exception of a patient with anterior mediastinal mass [17, 18]. Flexible fiberscopic intubation can be performed directly, using the special designed face mask [19] or supraglottic device as a conduit for flexible intubation [20]. It seems reasonable to preserve the spontaneous ventilation in patients with expected difficult airway. Supraglottic airway devices can resolve the situation or can be helpful as a route for fiberscopic intubation.

### 3.3. Unexpected Difficult Airway

**Figure 1:**
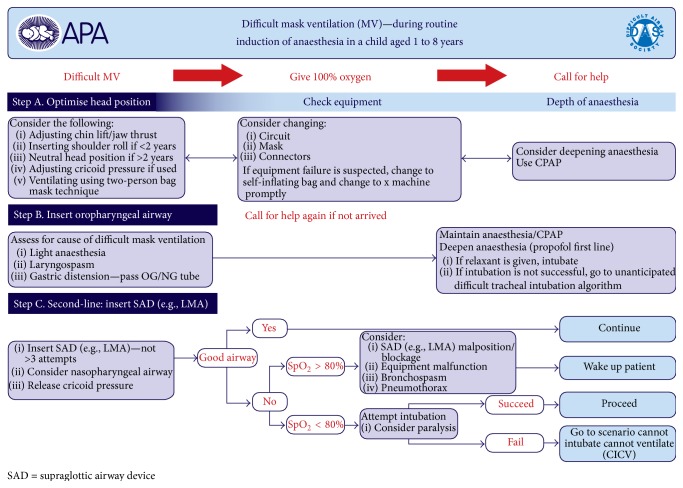
Guidelines for the management of difficult mask ventilation in children aged 1–8 years, published by DAS (Difficult Airway Society) at http://www.das.uk.com/guidelines/paediatric-difficult-airway-guidelines.

**Figure 2 fig2:**
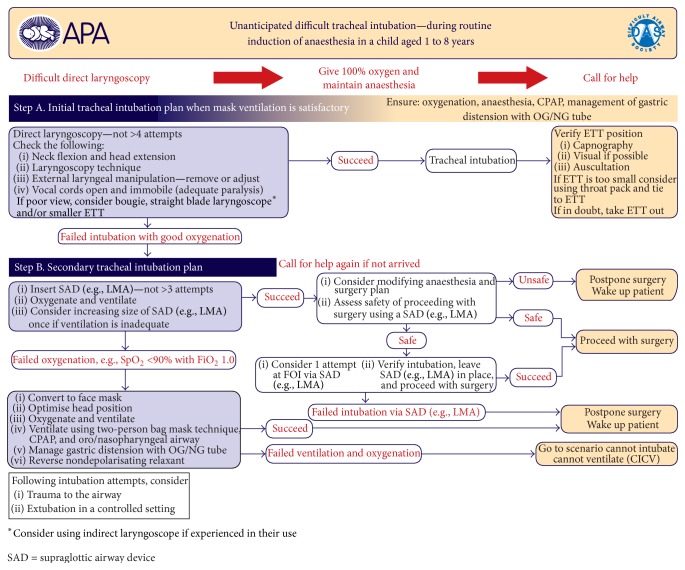
Guidelines for the management of unexpected difficult tracheal intubation in children aged 1–8 years, published by DAS (Difficult Airway Society) at http://www.das.uk.com/guidelines/paediatric-difficult-airway-guidelines.

**Figure 3:**
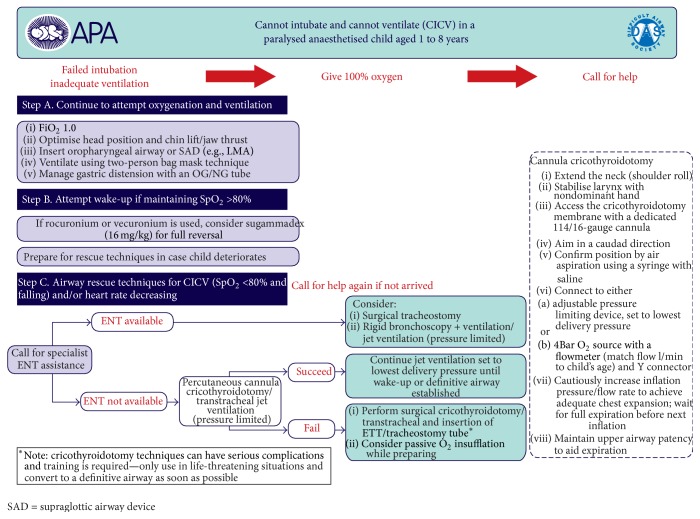
Guidelines for the management of CICV scenario in children aged 1–8 years, published by DAS (Difficult Airway Society) at http://www.das.uk.com/guidelines/paediatric-difficult-airway-guidelines.

There are currently published guidelines and reviews that summarize the recommendations in clinical situations of difficult mask ventilation, difficult tracheal intubation, and the cannot intubate cannot ventilate scenario in pediatric population [7, 10, 21]. Anatomically based problems can arise due to inadequate head position, airway collapse, inappropriate face mask handling, large tonsils, and/or adenoids. This can be overcome with proper positioning of the head, chin lift, jaw thrust, and two-hand manual ventilation via facemask [22]. However, functional airway obstructions are far more frequent and these can be caused by inadequate depth of anesthesia, laryngospasm, and opioid-induced glottic closure [14, 23, 24]. Laryngospasm is often treated with deepening the level of anesthesia although this may lead to significant hypotension in pediatric patients [25, 26]. Muscle paralysis for treating functional airway obstruction especially in case of cardiovascular instability is a more appropriate option [27]. The Difficult Airway Society (DAS) published guidelines for proceeding in emergency situations: unexpected difficult intubation during routine induction, difficult mask ventilation, and the cannot intubate cannot ventilate scenario in pediatric patients aged between 1 and 8 years (Figures 1 [Fig fig2]–3) [28]. This stepwise protocol is demonstrative and provides the proper directions for proceeding in life-threatening situations: difficult mask ventilation, unexpected difficult tracheal intubation, and the cannot intubate cannot ventilate scenario.

### 3.4. Cuffed or Uncuffed ETT?

Historically, uncuffed ETTs were used in pediatric patients under 8 years, to achieve a larger internal diameter of the tube, reducing flow resistance [29], and to minimalize possible oedema formation due to cuff caused mucosal damage. Currently, it is well documented that the narrowest part of the airway at the level of cricoid cartilage is elliptical. For this reason, there is the possibility of causing airway trauma also if the uncuffed tube with an acceptable leak pressure was used [12]. A higher incidence of laryngospasm with the use of uncuffed tubes has also been reported [12, 30]. The size of ETTs remains age-related [31]. It can be estimated using Cole's formula for uncuffed tube selection [32]: inner diameter (mm) = (16 + age)/4, although it has been reported that it can overestimate the actual tube size [33]. For cuffed tubes, Cole's formula results can be used, reduced by 0.5 or 1.0 mm [29], or another formula [34]: inner diameter (mm) = (age in years/4) + 3. The data show that accurately chosen and properly placed newly designed cuffed tubes (Microcuff) do not result in more airway-related complications than uncuffed ETT [34, 35] and can be used in infants [30]. One of the largest advantages of cuffed tubes is that they significantly reduce the exchange rate (from 25% to 2%) of ETTs after intubation in pediatric anesthesia [34]. No increase in morbidity has been reported with the latest cuffed ETT use in pediatric intensive care unit (ICU) patients [36] and according to the ILCOR (International Liaison Committee on Resuscitation) guidelines (2005), cuffed tubes are accepted as an alternative to uncuffed tubes [37]. Improper placement or excessive cuff pressure can lead to mucosal damage. It is highly recommended to periodically, ideally continuously monitor the cuff pressure to avoid potentially damaging pressures [38]. Newly designed (Microcuff) pediatric cuffed tubes are considered safe and effective in perioperative care for pediatric patient.

### 3.5. Rapid Sequence Induction (RSI)

RSI in adults is a standard procedure in patients with high risk of gastric aspiration (unfasted, trauma, GERD, gastroesophageal reflux, etc.). The most frequently used neuromuscular blocking agent during RSI is suxamethonium. The cricoid pressure (known as Sellick's Maneuver, SM) was subsequently added to the sequence to prevent gastric aspiration [39]. The cricoid pressure can also be effective in pediatric patients [40], but it can worsen intubation conditions [41–44]. It can also lead to a fall in lower esophageal sphincter tone [45]. The efficacy of this maneuver has been widely discussed over the past 20 years, with conflicting results. Another question is the proper performance of SM and the pressure that should be applied to the cricoid cartilage [43, 46, 47]. The data analysis showed that sufficient pressure to prevent aspiration is 10 N in awake patients and after induction the pressure should be raised to 30 N [48]. However, these data are derived from the adult population. During the past decade, SM has gradually been vanishing from routine anesthesiology practice. The results of its efficacy remain conflicting. It should be noticed that in Germany the routine use of SM in case of RSI in pediatric patients is no longer recommended [49] and in 2010 only 1.1% of pediatric anesthesiologists reported that they use SM during RSI in pediatric anesthesia [50].

Another conflicting issue is the use of suxamethonium in childhood. According to the Food and Drug Administration (FDA) recommendations, suxamethonium should be reserved only for emergency situations due to published adverse events and even deaths both in pediatric patients [51–53] and in adults [54–57]. Should we all abandon suxamethonium in pediatric anesthesia as it can be seen in some centers [58]? The authors recommend the well-known strategy “always have it (suxamethonium), never use it.” We can look at RSI from different points of view: do we need RSI? And do we have any alternative? The reported aspiration incidence in pediatric patients is low (0.4–1 per 1000), with a very low rate of serious complications [59, 60], and also we are able to measure gastric volume by ultrasound imaging [61, 62]. We definitely need to perform RSI in bowel obstruction or posttonsillectomy bleeding. However, some conditions routinely considered to be indications for RSI are today questionable as can be seen in a recent publication on gas induction in pyloromyotomy [63].

Do we have any alternative to suxamethonium? Rocuronium is the only neuromuscular blocking agent with comparable onset rapidity to suxamethonium. It provides good intubation conditions at 60 seconds [64]. The major break was the introduction of sugammadex, a chelating agent with high specificity for rocuronium reversal. Sugammadex is currently licensed in children over 2 years [65] but still not registered by the FDA (concerns about possible allergic reactions). Rocuronium and sugammadex can be used in difficult airway scenarios [66]; however, it should be noticed that successful reversal of neuromuscular blockade does not always lead to a successful end [67, 68], while the reason for CICV (cannot intubate cannot ventilate) can be multifactorial. The main RSI principle is the absence of manual hand-bag ventilation during the induction. The majority of children cannot be sufficiently preoxygenated before the induction and due to their low functional residual capacity and higher oxygen consumption, they will desaturate much faster than adults in the absence of ventilation and oxygenation. The classic-adult RSI will lead to hypoxia, bradycardia, and hypotension during induction [69]. Therefore, many authors recommend RSI adapted for childhood or “controlled RSI” [70] with deep anesthesia, muscle relaxation, and intermittent face mask ventilation [71–73].

### 3.6. Tracheal Intubation versus Laryngeal Mask

Laryngeal masks (LMs) are today commonly used in routine pediatric anesthesia practice in a whole spectrum of surgical procedures [74–76]. Laryngeal mask can be effectively used in difficult airway management [77] and also in a large number of elective procedures [78, 79]. The LM use can lead to significant reduction of postoperative desaturation, laryngospasm, and cough and reduction in postanesthetic unit stay compared to ETTs [80]. The reason for the widespread use and high popularity of LMs by anesthesiologists may be the low failure rate and rapid learning curve [78, 79]. In case of insufficient seal, reposition, reinsertion or head flection, and rotation can lead to minimizing the air leak [81, 82]. However, even achieving a good seal does not guarantee the proper position of the LM [83]. For this reason, it is highly recommended to monitor cuff pressure during the anesthesia [84]. Possible gastric acid reflux is a question when using LM. The rate of reported aspiration appears to be very low although silent gastroesophageal reflux can often occur. It appears that, in reported cases, the diagnosis of reflux had no clinical consequences and compared to facemask ventilation and anesthesia with intubation, the incidence was similar [85, 86]. Laryngeal masks have been used for airway management during adenotonsillectomy, tonsillectomy, and adenoidectomy [87], laparoscopic surgeries (with comparable intragastric pressure to ETT [88]), during fibreoptic bronchoscopy [89, 90], eye surgery [91], during difficult airway management as a conduit for ETT placement [92, 93], and during resuscitation (also in neonates) with no documented difference in outcome compared to ETT use. LMs have earned their reputation for superior performance, simplicity, and low rate of failure also in pediatric anesthesia. Limitation of LMs can be seen in conditions such as Pulmonary alveolar Proteinosis (PaP) where the lung separation for invasive treatment is inevitable [94]. LMs have saved many lives and anesthesiologists' careers. We must also bear in mind, however, the limitations of this device (leak pressure, failure rate, and regurgitation risk) and the risk versus benefit ratio should always be considered in deciding between ETT and LM for pediatric patient's airway management. These data should not be interpreted as the uselessness of ETT and RSI in patients with high risk of aspiration (unfasted, major trauma, etc.), because in light of current EBM data it would be non lege artis practice.

### 3.7. When to Extubate the Pediatric Patient?

**Figure 4:**
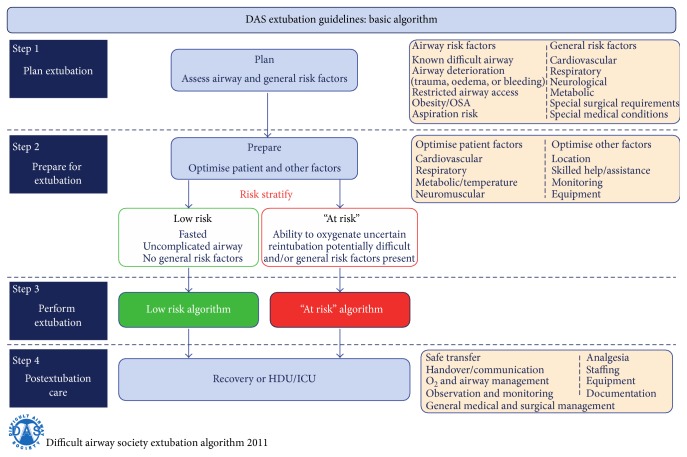
Guidelines for the management of tracheal extubation, basic algorithm, published by DAS (Difficult Airway Society) at http://www.das.uk.com/guidelines/paediatric-difficult-airway-guidelines.

**Figure 5 fig5:**
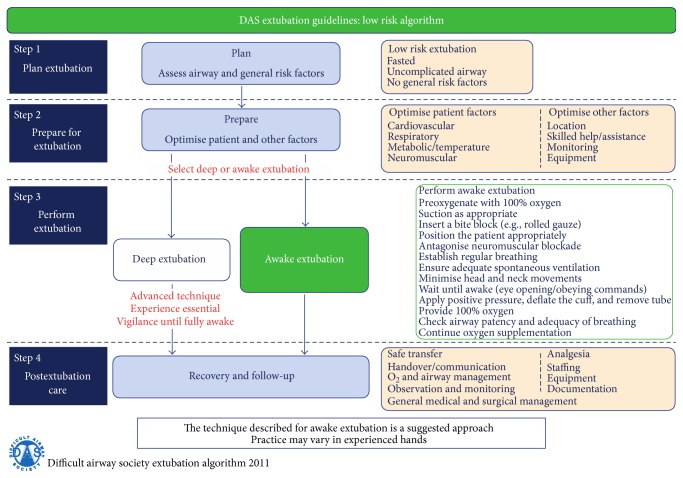
Guidelines for the management of tracheal extubation, low risk algorithm, published by DAS (Difficult Airway Society) at http://www.das.uk.com/guidelines/paediatric-difficult-airway-guidelines.

**Figure 6:**
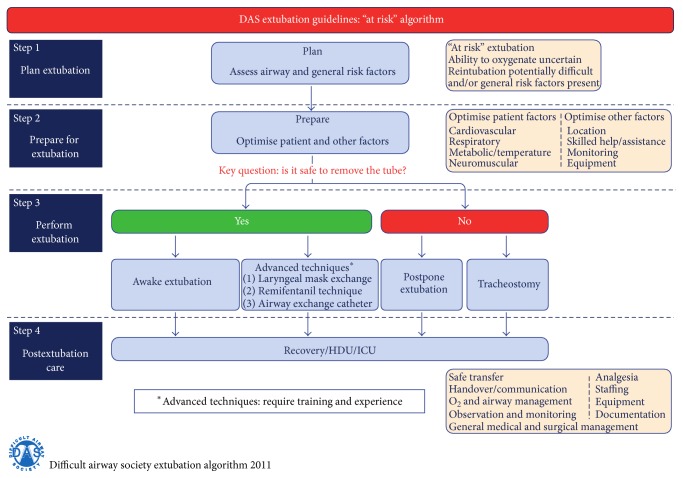
Guidelines for the management of tracheal extubation, high risk algorithm, published by DAS (Difficult Airway Society) at http://www.das.uk.com/guidelines/paediatric-difficult-airway-guidelines.

The emergence from anesthesia is another risky situation during the perioperative period. The anesthesiologist should decide whether to extubate the child in deep anesthesia or awake with sufficient spontaneous ventilation or whether to proceed with the mechanical ventilation in ICU because of surgery duration, hypothermia, hemodynamic instability, respiratory distress, massive blood loss, and other conditions should be considered prior to extubation. The operator must consider two questions: intubation conditions and the risk of aspiration. In the case of difficult airway and in patients with high risk of aspiration, it is generally recommended to extubate them when awake with sufficient spontaneous ventilation with appropriate protective airway reflexes. In pediatric patients, it has been reported that the routine practice is extubation during deep anesthesia [95]. This can lead to minimizing cardiovascular system stimulation and reducing the incidence of cough; however, some data reported a higher incidence of respiratory complications with this practice [96]. Extubation at the moment of end-inspiration can minimize the risk of laryngospasm [97] and experienced anesthesiologist is associated with lower risk of laryngospasm [98]. In conclusion, the difference in both practices (awake or in deep anesthesia intubation) is not associated with impact on outcome [99]. The main exception is the child with difficult airway and the child with high risk of aspiration, where the consensus is clear, extubating them awake with sufficient spontaneous ventilation. Compendious extubation guidelines have been published by DAS (Figures 4 [Fig fig5]–6) [28]. The guideline is primarily for adult patients; however, with respect to differences in pediatric anesthesia, they could be implemented in pediatric extubation management.

## 4. Conclusion

The majority of difficult airway in childhood can be predicted and the best method for prediction seems to be the combination of clinical examination with predictors with good performance: mandibular protrusion, Mallampati's classification, movement of atlantooccipital joint, and thyromental distance. In case of anticipated difficult airway, it is advisable to preserve spontaneous ventilation. The classic RSI is not suitable for children and the mild (airway pressures under 20 cm H_2_O) hand-bag ventilation is considered a safe method during pediatric RSI that provides oxygenation and minimizes possible hypoxia. Although we can see an increasing number of RCTs dedicated to pediatric airway management, there is still need to perform well designed large RCTs in pediatric subpopulation to formulate the airway management guidelines based on pediatric EBM data.

## References

[1] Litman R. S., Weissend E. E., Shibata D., Westesson P.-L. (2003). Developmental changes of laryngeal dimensions in unparalyzed, sedated children. *Anesthesiology*.

[2] Karkouti K., Rose D. K., Wigglesworth D., Cohen M. M. (2000). Predicting difficult intubation: a multivariable analysis. *Canadian Journal of Anesthesia*.

[3] Frei F. J., Ummenhofer W. (1996). Difficult intubation in paediatrics. *Paediatric Anaesthesia*.

[4] Heinrich S., Birkholz T., Ihmsen H., Irouschek A., Ackermann A., Schmidt J. (2012). Incidence and predictors of difficult laryngoscopy in 11.219 pediatric anesthesia procedures. *Paediatric Anaesthesia*.

[5] Heinrich S., Birkholz T., Irouschek A., Ackermann A., Schmidt J. (2013). Incidences and predictors of difficult laryngoscopy in adult patients undergoing general anesthesia: a single-center analysis of 102,305 cases. *Journal of Anesthesia*.

[6] Krishna S. G., Tobias J. D. (2014). An update on airway management in infants and children. *Anaesthesia, Pain & Intensive Care*.

[7] Weiss M., Engelhardt T. (2010). Proposal for the management of the unexpected difficult pediatric airway. *Paediatric Anaesthesia*.

[8] Russoa S. G., Becke K. (2015). Expected difficult airway in children. *Current Opinion in Anesthesiology*.

[9] Ohkawa S. (2005). Incidence of difficult intubation in pediatric population. *Anesthesiology*.

[10] Mirghassemi A., Soltani A. E., Abtahi M. (2011). Evaluation of laryngoscopic views and related influencing factors in a pediatric population. *Paediatric Anaesthesia*.

[11] Harless J., Ramaiah R., Bhananker S. M. (2014). Pediatric airway management. *International Journal of Critical Illness and Injury Science*.

[12] Nargozian C. (2004). The airway in patients with craniofacial abnormalities. *Paediatric Anaesthesia*.

[13] Sims C., von Ungern-Sternberg B. S. (2012). The normal and the challenging pediatric airway. *Paediatric Anaesthesia*.

[14] Jimenez N., Posner K. L., Cheney F. W., Caplan R. A., Lee L. A., Domino K. B. (2007). An update on pediatric anesthesia liability: a closed claims analysis. *Anesthesia and Analgesia*.

[15] Bhananker S. M., Ramamoorthy C., Geiduschek J. M. (2007). Anesthesia-related cardiac arrest in children: update from the pediatric perioperative cardiac arrest registry. *Anesthesia and Analgesia*.

[16] Sunder R. A., Haile D. T., Farrell P. T., Sharma A. (2012). Pediatric airway management: current practices and future directions. *Paediatric Anaesthesia*.

[17] Hack H. A., Wright N. B., Wynn R. F. (2008). The anaesthetic management of children with anterior mediastinal masses. *Anaesthesia*.

[18] Stricker P. A., Gurnaney H. G., Litman R. S. (2010). Anesthetic management of children with an anterior mediastinal mass. *Journal of Clinical Anesthesia*.

[19] Frei F. J., Wengen D. F., Rutishauser M., Ummenhofer W. P. (1995). The airway endoscopy mask: useful device for fibreoptic evaluation and intubation of the paediatric airway. *Paediatric Anaesthesia*.

[20] Weiss M., Mauch J., Becke K., Schmidt J., Jöhr M. (2009). Fibre optic-assisted endotracheal intubation through the laryngeal mask in children. *Anaesthesist*.

[21] Black A. E., Flynn P. E. R., Smith H. L., Thomas M. L., Wilkinson K. A. (2015). Development of a guideline for the management of the unanticipated difficult airway in pediatric practice. *Paediatric Anaesthesia*.

[22] Engelhardt T., Weiss M. (2012). A child with a difficult airway: what do I do next?. *Current Opinion in Anaesthesiology*.

[23] Mamie C., Habre W., Delhumeau C., Argiroffo C. B., Morabia A. (2004). Incidence and risk factors of perioperative respiratory adverse events in children undergoing elective surgery. *Paediatric Anaesthesia*.

[24] Murat I., Constant I., Maud'Huy H. (2004). Perioperative anaesthetic morbidity in children: a database of 24 165 anaesthetics over a 30-month period. *Paediatric Anaesthesia*.

[25] Lerman J., Houle T. T., Matthews B. T., Houck J., Burrows F. A. (2009). Propofol for tracheal intubation in children anesthetized with sevoflurane: a dose-response study. *Paediatric Anaesthesia*.

[26] Nafiu O. O., Kheterpal S., Morris M., Reynolds P. I., Malviya S., Tremper K. K. (2009). Incidence and risk factors for preincision hypotension in a noncardiac pediatric surgical population. *Paediatric Anaesthesia*.

[27] Calder I., Yentis S. M. (2008). Could ‘safe practice’ be compromising safe practice? Should anaesthetists have to demonstrate that face mask ventilation is possible before giving a neuromuscular blocker?. *Anaesthesia*.

[28] Popat M., Mitchell V., Dravid R., Patel A., Swampillai C., Higgs A. (2012). Difficult Airway Society Guidelines for the management of tracheal extubation. *Anaesthesia*.

[29] Brambrink A. M., Meyer R. R. (2002). Management of the paediatric airway: new developments. *Current Opinion in Anaesthesiology*.

[30] Fine G. F., Fertal K., Motoyama E. K. (2000). The effectiveness of controlled ventilation using cuffed versus uncuffed ETT in infants. *Anesthesiology*.

[31] King B. R., Baker M. D., Braitman L. E., Seidl-Friedman J., Schreiner M. S. (1993). Endotracheal tube selection in children: a comparison of four methods. *Annals of Emergency Medicine*.

[32] Cole F. (1957). Pediatric formulas for the anesthesiologist.. *The American Journal of Diseases of Children*.

[33] Hofer C. K., Ganter M., Tucci M., Klaghofer R., Zollinger A. (2002). How reliable is length-based determination of body weight and tracheal tube size in the paediatric age group? The Broselow tape reconsidered. *British Journal of Anaesthesia*.

[34] Khine H. H., Corddry D. H., Kettrick R. G. (1997). Comparison of cuffed and uncuffed endotracheal tubes in young children during general anesthesia. *Anesthesiology*.

[35] James I. (2001). Cuffed tubes in children. *Paediatric Anaesthesia*.

[36] Newth C. J. L., Rachman B., Patel N., Hammer J. (2004). The use of cuffed versus uncuffed endotracheal tubes in pediatric intensive care. *Journal of Pediatrics*.

[37] The International Liaison Committee on Resuscitation (ILCOR) (2005). Consensus science with treatment recommendations for paediatric and neonatal patients: paediatric basic and advanced life support. *Paediatrics*.

[38] Tobias J. D., Schwartz L., Rice J., Jatana K., Kang D. R. (2012). Cuffed endotracheal tubes in infants and children: should we routinely measure the cuff pressure?. *International Journal of Pediatric Otorhinolaryngology*.

[39] Sellick B. A. (1961). Cricoid pressure to control regurgitation of stomach contents during the induction of anaesthesia. *The Lancet*.

[40] Salem M. R., Wong A. Y., Fizzotti G. F. (1972). Efficacy of cricoid pressure in preventing aspiration of gastric contents in paediatric patients. *British Journal of Anaesthesia*.

[41] Brock-Utne J. G. (2002). Is cricoid pressure necessary?. *Paediatric Anaesthesia*.

[42] Ellis D. Y., Harris T., Zideman D. (2007). Cricoid pressure in emergency department rapid sequence tracheal intubations: a risk-benefit analysis. *Annals of Emergency Medicine*.

[43] Turgeon A. F., Nicole P. C., Trépanier C. A., Marcoux S., Lessard M. R. (2005). Cricoid pressure does not increase the rate of failed intubation by direct laryngoscopy in adults. *Anesthesiology*.

[44] Noguchi T., Koga K., Shiga Y., Shigematsu A. (2003). The gum elastic bougie eases tracheal intubation while applying cricoid pressure compared to a stylet. *Canadian Journal of Anesthesia*.

[45] Tournadre J.-P., Chassard D., Berrada K. R., Boulétreau P. (1997). Cricoid cartilage pressure decreases lower esophageal sphincter tone. *Anesthesiology*.

[46] Wraight W. J., Chamney A. R., Howells T. H. (1983). The determination of an effective cricoid pressure. *Anaesthesia*.

[47] Vanner R. G., O'Dwyer J. P., Pryle B. J., Reynolds F. (1992). Upper oesophageal sphincter pressure and the effect of cricoid pressure. *Anaesthesia*.

[48] Vanner R. G., Asai T. (1999). Safe use of cricoid pressure. *Anaesthesia*.

[49] Schmidt J., Strauß J. M., Becke K., Giest J., Schmitz B. (2007). Handlungsempfehlung zur Rapid-Sequence-Induction im Kindesalter. *Anäst Intensivmed*.

[50] Walker R. W., Ravi R., Haylett K. (2009). Effect of cricoid force on airway calibre in children: a bronchoscopic assessment. *British Journal of Anaesthesia*.

[51] Rosenberg H., Gronert G. A. (1992). Intractable cardiac arrest in children given succinylcholine. *Anesthesiology*.

[52] Wang J. M., Stanley T. H. (1986). Duchenne muscular dystrophy and malignant hyperthermia: two case reports. *Canadian Anaesthetists Society Journal*.

[53] Wu C. C., Tseng C. S., Shen C. H., Yang T. C., Chi K. P., Ho W. M. (1998). Succinylcholine induced cardiac arrest in children with unsuspected Beck muscular dystrophy: a case report. *Acta Anaesthesiologica Sinica*.

[54] Sato K., Nishiwaki K., Kuno N. (2000). Unanticipated hyperkalemia following succinylcholine administration in prolonged immobilized parturients treated with magnesium and ritodrine. *Anesthesiology*.

[55] Bauer S. J., Orio K., Adams B. D. (2005). Succinylcholine induced masseter spasm during rapid sequence intubation may require a surgical airway: case report. *Emergency Medicine Journal*.

[56] Gill M., Graeme K., Guenterberg K. (2005). Masseter spasm after succinylcholine administration. *Journal of Emergency Medicine*.

[57] Onyeka T. C. U. (2010). Masseter muscle rigidity: atypical malignant hyperthermia presentation or isolated event?. *Saudi Journal of Anaesthesia*.

[58] Sparr H. J., Jöhr M. (2002). Succinylcholin update. *Anaesthesist*.

[59] Cubillos J., Tse C., Chan V. W. S., Perlas A. (2012). Bedside ultrasound assessment of gastric content: an observational study. *Canadian Journal of Anesthesia*.

[60] Schmitz A., Thomas S., Melanie F. (2012). Ultrasonographic gastric antral area and gastric contents volume in children. *Paediatric Anaesthesia*.

[61] Warner M. A., Warner M. E., Warner D. O., Warner L. O., Warner E. J. (1999). Perioperative pulmonary aspiration in infants and children. *Anesthesiology*.

[62] Borland L. M., Sereika S. M., Woelfel S. K. (1998). Pulmonary aspiration in pediatric patients during general anesthesia: incidence and outcome. *Journal of Clinical Anesthesia*.

[63] Scrimgeour G. E., Leather N. W., Perry R. S., Pappachan J. V., Baldock A. J., Bosenberg A. (2015). Gas induction for pyloromyotomy. *Pediatric Anesthesia*.

[64] Perry J. J., Lee J. S., Sillberg V. A. H., Wells G. A. (2008). Rocuronium versus succinylcholine for rapid sequence induction intubation. *Cochrane Database of Systematic Reviews*.

[65] Paediatric Formulary Committee (2014). *BNF for Children*.

[66] Desforges J. C. W., McDonnell N. J. (2011). Sugammadex in the management of a failed intubation in a morbidly obese patient. *Anaesthesia and Intensive Care*.

[67] Curtis R., Lomax S., Patel B. (2012). Use of sugammadex in a ‘can't intubate, can't ventilate’ situation. *British Journal of Anaesthesia*.

[68] Kyle B. C., Gaylard D., Riley R. H. (2012). A persistent ‘can't intubate, can't oxygenate’ crisis despite rocuronium reversal with sugammadex. *Anaesthesia and Intensive Care*.

[69] Gencorelli F. J., Fields R. G., Litman R. S. (2010). Complications during rapid sequence induction of general anesthesia in children: a benchmark study. *Paediatric Anaesthesia*.

[70] Engelhardt T., Strachan L., Johnston G. (2001). Aspiration and regurgitation prophylaxis in paediatric anaesthesia. *Paediatric Anaesthesia*.

[71] Neuhaus D., Schmitz A., Gerber A., Weiss M. (2013). Controlled rapid sequence induction and intubation—an analysis of 1001 children. *Paediatric Anaesthesia*.

[72] Fields R. G., Gencorelli F. J., Litman R. S. (2010). Anesthetic management of the pediatric bleeding tonsil. *Paediatric Anaesthesia*.

[73] Eich C., Weiss M., Neuhaus D., Strauß J., Jöhr M., Becke K. (2010). Incidence of complications associated with rapid sequence induction (RSI) in children—it is a matter of age and technique. *Paediatric Anaesthesia*.

[74] Boehringer L. A., Bennie R. E., Ferson D. Z., Brimacombe J. R., Brain A. I. J. (1998). Laryngeal mask airway and the pediatric patient. *The Laryngeal Mask Airway. International Anesthesiology Clinics*.

[75] Landsman I. S., Borland L. W. (1997). The laryngeal mask airway. *Airway Management in Pediatric Anesthesia. International Anesthesiology Clinics*.

[76] Goldmann K. (2006). Recent developments in airway management of the paediatric patient. *Current Opinion in Anaesthesiology*.

[77] White M. C., Cook T. M., Stoddart P. A. (2009). A critique of elective pediatric supraglottic airway devices. *Paediatric Anaesthesia*.

[78] Lopez-Gil M., Brimacombe J., Alvarez M. (1996). Safety and efficacy of the laryngeal mask airway. A prospective survey of 1400 children. *Anaesthesia*.

[79] Lopez-Gil M., Brimacombe J., Cebrian J., Arranz J. (1996). Laryngeal mask airway in pediatric practice: a prospective study of skill acquisition by anesthesia residents. *Anesthesiology*.

[80] Luce V., Harkouk H., Brasher C. (2014). Supraglottic airway devices vs tracheal intubation in children: a quantitative meta-analysis of respiratory complications. *Paediatric Anaesthesia*.

[81] Xue F. S., Mao P., Liu H. P. (2008). The effects of head flexion on airway seal, quality of ventilation and orogastric tube placement using the ProSeal laryngeal mask airway. *Anaesthesia*.

[82] Choi W. J., Kim Y. H. (2007). The influence of head rotation on ProSeal laryngeal mask airway sealing during paediatric myringotomy. *Anaesthesia and Intensive Care*.

[83] Inagawa G., Okuda K., Miwa T., Hiroki K. (2000). Good airway seal does not imply the correct position of laryngeal mask airway in pediatric patients. *Anesthesia & Analgesia*.

[84] Hockings L., Heaney M., Chambers N. A., Erb T. O., Von Ungern-Sternberg B. S. (2010). Reduced air leakage by adjusting the cuff pressure in pediatric laryngeal mask airways during spontaneous ventilation. *Paediatric Anaesthesia*.

[85] Cebrián J., Avellanal M., Morales J. L. (2000). Continuous monitoring of oesophageal pH during general anaesthesia with laryngeal mask airway in children. *Paediatric Anaesthesia*.

[86] Özlü O., Türker A. K., Özgün G., Soykan I. (2001). Distal oesophageal pH measurement in children during general anaesthesia using the laryngeal mask airway, tracheal tube and face mask. *Paediatric Anaesthesia*.

[87] Sierpina D. I., Chaudhary H., Walner D. L. (2012). Laryngeal mask airway versus endotracheal tube in pediatric adenotonsillectomy. *The Laryngoscope*.

[88] Ozdamar D., Güvenç B. H., Toker K., Solak M., Ekingen G. (2010). Comparison of the effect of LMA and ETT on ventilation and intragastric pressure in pediatric laparoscopic procedures. *Minerva Anestesiologica*.

[89] Lesmes C., Siplovich L., Katz Y. (2000). Fiberoptic bronchoscopy in children using the laryngeal mask airway. *Pediatric Surgery International*.

[90] Nussbaum E., Zagnoev M. (2001). Pediatric fiberoptic bronchoscopy with a laryngeal mask airway. *Chest*.

[91] Duman A., Ögün C. Ö., Ökesli S. (2001). The effect on intraocular pressure of tracheal intubation or laryngeal mask insertion during sevoflurane anaesthesia in children without the use of muscle relaxants. *Paediatric Anaesthesia*.

[92] Walker R. W. M. (2001). Management of the difficult airway in children. *Journal of the Royal Society of Medicine*.

[93] Michalek P., Donaldson W., Theiler L. (2013). The use of the i-gel in anaesthesia—facts and fiction in 2013. *Trends in Anaesthesia and Critical Care*.

[94] Vymazal T., Krecmerova M. (2015). Respiratory strategies and airway management in patients with pulmonary alveolar proteinosis: a review. *BioMed Research International*.

[95] Tsui B. C. H., Wagner A., Cave D., Elliott C., El-Hakim H., Malherbe S. (2004). The incidence of laryngospasm with a ‘no touch’ extubation technique after Tonsillectomy and adenoidectomy. *Anesthesia & Analgesia*.

[96] Asai T., Koga K., Vaughan R. S. (1998). Respiratory complications associated with tracheal intubation and extubation. *British Journal of Anaesthesia*.

[97] Miller K. A., Harkin C. P., Bailey P. L. (1995). Postoperative tracheal extubation. *Anesthesia and Analgesia*.

[98] Al-alami A. A., Zestos M. M., Baraka A. S. (2009). Pediatric laryngospasm: prevention and treatment. *Current Opinion in Anaesthesiology*.

[99] Patel R. I., Hannallah R. S., Norden J., Casey W. F., Verghese S. T. (1991). Emergence airway complications in children: a comparison of tracheal extubation in awake and deeply anesthetized patients. *Anesthesia and Analgesia*.

